# Evolutionary Dynamics of the Repetitive DNA in the Karyotypes of *Pipa carvalhoi* and *Xenopus tropicalis* (Anura, Pipidae)

**DOI:** 10.3389/fgene.2020.00637

**Published:** 2020-07-21

**Authors:** Michelle Louise Zattera, Camilla Borges Gazolla, Amanda de Araújo Soares, Thiago Gazoni, Nicolas Pollet, Shirlei Maria Recco-Pimentel, Daniel Pacheco Bruschi

**Affiliations:** ^1^Programa de Pós-Graduação em Genética (PPG-GEN), Universidade Federal do Paraná (UFPR), Curitiba, Brazil; ^2^Universidade Estadual Paulista (Unesp), Campus Rio Claro, Rio Claro, Brazil; ^3^Laboratoire Evolution Genomes Comportement Ecologie, CNRS, IRD, Université Paris-Saclay, Gif-sur-Yvette, France; ^4^Departamento de Biologia Estrutural e Funcional, Universidade Estadual de Campinas (UNICAMP), Campinas, Brazil

**Keywords:** Pipidae, multigene family, microsatellite, chromosomal evolution, histone H3

## Abstract

The large amphibian genomes contain numerous repetitive DNA components that have played an important role in the karyotypic diversification of this vertebrate group. Hypotheses based on the presumable primitive karyotype (2n = 20) of the anurans of the family Pipidae suggest that they have evolved principally through intrachromosomal rearrangements. *Pipa* is the only South American pipid, while all the other genera are found in Africa. The divergence of the South American lineages from the African ones occurred at least 136 million years ago and is thought to have had a strong biogeographic component. Here, we tested the potential of the repetitive DNA to enable a better understanding of the differentiation of the karyotype among the family Pipidae and to expand our capacity to interpret the chromosomal evolution in this frog family. Our results indicate a long history of conservation in the chromosome bearing the H3 histone locus, corroborating inferences on the chromosomal homologies between the species in pairs 6, 8, and 9. The chromosomal distribution of the microsatellite motifs also provides useful markers for comparative genomics at the chromosome level between *Pipa carvalhoi* and *Xenopus tropicalis*, contributing new insights into the evolution of the karyotypes of these species. We detected similar patterns in the distribution and abundance of the microsatellite arrangements, which reflect the shared organization in the terminal/subterminal region of the chromosomes between these two species. By contrast, the microsatellite probes detected a differential arrangement of the repetitive DNA among the chromosomes of the two species, allowing longitudinal differentiation of pairs that are identical in size and morphology, such as pairs 1, 2, 4, and 5. We also found evidence of the distinctive composition of the repetitive motifs of the centromeric region between the species analyzed in the present study, with a clear enrichment of the (CA) and (GA) microsatellite motifs in *P. carvalhoi*. Finally, microsatellite enrichment in the pericentromeric region of chromosome pairs 6, 8, and 9 in the *P. carvalhoi* karyotype, together with interstitial telomeric sequences (ITS), validate the hypothesis that pericentromeric inversions occurred during the chromosomal evolution of *P. carvalhoi* and reinforce the role of the repetitive DNA in the remodeling of the karyotype architecture of the Pipidae.

## Introduction

Amphibians are a diverse and abundant class of vertebrates that provide an important model for studies in evolutionary genetics (Voss et al., 2011; Session et al., 2016). Amphibian genomes are always used as examples of the phenomenon known as the C-value paradox, given that they are relatively large (Gregory, 2005), in general mainly because of their considerable content of repetitive DNA (Sun et al., 2015; Liedtke et al., 2018; Sclavi and Herrick, 2018).

In addition to its structural role in the eukaryote chromosome, the repetitive fraction of the genome also plays a central role in the stability of the chromosome, the cell cycle, and the regulation of gene expression and is an important substrate for genome evolution (Foulongne-Oriol et al., 2013; Biscotti et al., 2015; Liu et al., 2019). Macrostructurally, sequences of repetitive DNA are involved directly or indirectly in the chromosomal rearrangement events (i.e., deletions, duplications, inversions, and translocations) that are responsible for the significant karyotypic variation observed during the evolution of many groups of organisms (Kidwell, 2002; Feschotte and Pritham, 2007; Cazaux et al., 2011; González and Petrov, 2012; Prakhongcheep et al., 2017; Supiwong et al., 2019).

The mechanisms involved in the evolution of repetitive DNA operate at an intragenomic level and are directly related to the organization of these repetitive sequences in the chromosomes. These repetitive sequences can be divided into two major groups: the dispersed DNA (transposable elements) and DNA sequences arranged *in tandem*, such as microsatellite, mini-satellite, and satellite DNA (Kidwell, 2002; Böhne et al., 2008; Biscotti et al., 2015; Mlinarec et al., 2019). Satellite DNA (including micro- and minisatellites) is made up of systematic *in tandem* repeats that favor the occurrence of chromosomal rearrangements, ectopic recombination, and genic conversion (Schweizer and Loidl, 1987; Louzada et al., 2020). Microsatellite repeats are a good example here, being formed by short (2–7 bases) sequences with a large number of repetitions (Yashima and Innan, 2016). These sequences present high rates of variation and may be a component of either the heterochromatin (Kubat et al., 2008; Martins et al., 2013; Lopes et al., 2014) or the euchromatin (Kuhn et al., 2011; Pavlek et al., 2015; Ruiz-Ruano et al., 2016; Pita et al., 2017).

By contrast, the multigene families that encode fundamental molecules (e.g., histone genes, rDNAs, and non-codifying nuclear RNAs – snoRNAs) are subject to strong selective pressures and often maintain a conserved nucleotide sequence and chromosome position over the evolutionary history of a lineage (Piontkivska et al., 2002; Piscor and Parise-Maltempi, 2016). This makes these gene families excellent chromosomal markers for the comparative study of the organization of the genome in different species (Cabrero et al., 2009; Cabral-De-Mello et al., 2011; Anjos et al., 2015).

There is considerable evidence that the chromosomal organization of different repetitive DNA classes is conserved during the karyotype evolution of closely related species (Ruiz-Ruano et al., 2016) and that the study of this DNA may provide important insights for the understanding of the chromosomal evolution of these groups. From this perspective, the amphibians of the family Pipidae are an interesting group for the analysis of chromosomal evolution.

The family Pipidae includes 41 species distributed in four genera, *Hymenochirus, Pseudhymenochirus*, *Pipa*, and *Xenopus* (Frost, 2020). *Pipa* is restricted to Central and South America, while the other genera are distributed in Sub-Saharan Africa (Frost, 2020). A recent phylogenetic reconstruction defined *Pipa* as the sister group of the African genera [(*Xenopus* + *Silurana*) + (*Hymenochirus* + *Pseudhymenochirus*)] (Irisarri et al., 2011). Despite this, the diversification of the African and South American lineages is still the subject of controversy, given that the fossil, morphological, and molecular data are contradictory (Cannatella, 2015). This hampers the interpretation of the biogeographic history of the Pipidae and the phylogenetic relationship between *Pipa* and the other extant genera (i.e., whether *Pipa* + *Xenopus* or *Pipa* + *Hymenochirus*). Thus, the biogeographic scenario that accounts for the diversification of these lineages remains unsolved.

The 2*n* = 20 karyotype is a putative plesiomorphic condition in the family Pipidae (Morescalchi, 1981; Mezzasalma et al., 2015; Zattera et al., 2019). This condition can be observed in the karyotypes of *Pipa carvalhoi*, *Pseudhymenochirus merli*, *Hymenochirus boettgeri*, and in *Xenopus tropicalis* (Tymowska and Fischberg, 1973; Mezzasalma et al., 2015; Zattera et al., 2019). In the genus *Xenopus*, in particular, two well-supported clades have been recognized (Evans et al., 2019). One clade, known as the *X. tropicalis* group, includes the species with 2*n* = 20 chromosomes and the polyploid species derived from this basic type [2*n* = 4*x* = 40: *X. calcaratus*; *X. epitropicalis*; X. *mellotropicalis*] (Evans et al., 2015). The second clade, known as the *Xenopus laevis* group, encompasses 25 species, with diploid numbers ranging from 4*n* = 36 to 12*n* = 108, resulting from a series of independent allopolyploidization events beginning with an ancestral karyotype of 2*n* = 18 chromosomes (see the list to Zattera et al., 2019). In this case, the primitive 2*n* = 18 karyotype appears to have been derived from the fusion of chromosomes 9 + 10 in the ancestral 2*n* = 20 karyotype (Mezzasalma et al., 2015; Session et al., 2016).

If this hypothesis is true, the diversification of the pipid karyotype would be due primarily to intrachromosomal rearrangements (see Mezzasalma et al., 2015). As the morphology of pairs 1, 2, 3, and 4 of the pipid karyotype is conserved, as it is in *Rinophrynus dorsalis* (sister-group of the Pipidae), the use of the repetitive DNA as a probe in FISH assays should enable the identification of karyological differences imperceptible by classical chromosome markers. The presumable primitive karyotype of the Pipidae, for example (which is conserved in *X. tropicalis*), indicates the occurrence of five pericentromeric inversions (in pairs 3, 6, 8, 9, and 10) in the *P. carvalhoi* karyotype (Mezzasalma et al., 2015) and three inversions (in pairs 6, 8 and 10) in *P. merli* (Mezzasalma et al., 2015). Recently, Zattera et al. (2019) validated some of these pericentromeric inversions in *P. carvalhoi* by FISH assays, as confirmed by the presence of the interstitial telomeric sequences (ITS) in the homologs of pairs 6, 8, and 9.

This hypothesis of interspecific chromosomal homeologies in the Pipidae is based primarily on the morphology and centromeric position, given the lack of evidence on more detailed karyotype features in the pipids. This paucity of informative markers limits comparisons among the karyotypes, which precludes an adequate interpretation of the chromosomal evolution of this family. *P. carvalhoi* and *X. tropicalis* share the same diploid number (20 chromosomes) but diverged at least 136 million years ago (Cannatella, 2015). Here, we tested the potential of the repetitive DNA to enable a better understanding of the differentiation of the karyotype among the family Pipidae and to expand our capacity to interpret the chromosomal evolution in this frog family.

For this, we compared the chromosomal arrangement of seven microsatellite motifs and one multigene family (the histone H3 gene) in the karyotypes of *P. carvalhoi* and *X. tropicalis*. We verified the conservation of the chromosomal organization of these repeats to discuss their importance in the evolution and function of these genomes.

## Materials and Methods

### Chromosomal Preparations

The five specimens of *P. carvalhoi* analyzed in the present study were collected in Buíque, Pernambuco, Brazil (08°37′23″ S, 37°09′21″ W), and in all experiments at least ten metaphases were analyzed in each slide. Specimen collection was authorized by SISBIO/Chico Mendes Institute for Biodiversity Conservation (protocol 55481-1), and the specimens were deposited in the Natural History Museum of the Federal University of Alagoas (MHN-UFAL) in Maceió, Brazil. The *P. carvalhoi* specimens were injected intraperitoneally with 2% colchicine (0.02 mL/g of the animal) for 4 h to obtain the chromosome preparations, with the suspensions of the intestine and testicles being obtained following the protocol of King and Rofe (1976) and Schmid (1978). Samples of Speedy cell suspensions of *X. tropicalis* were obtained through the culture of the fibroblast cells in the laboratory of Dr. Malcolm A. Ferguson-Smith of the Department of Veterinary Medicine at the University of Cambridge (Sinzelle et al., 2012).

### Preparation and Chromosomal Mapping of the Histone H3 Probe

A 400 base pair (bp) fragment of the histone H3 gene was synthesized from *P. carvalhoi* DNA by PCR using the primers H3-F (5′-ATGGCTCGTACCAAGCAGACVGC 3′) and H3-R (5′ ATATCCTTRGGCATRATRGTGAC 3′) following Colgan et al. (1998). The PCR product was purified using the EasyPure Quick Gel Extraction kit (PROMEGA), following the manufacturer’s recommendations, and inserted in the pJET 1.2/blunt cloning vector. The recombinant DNA was used to transform *Escherichia coli TOP10* cells. The recombinant clones were grown for plasmid DNA mini-prep extraction as described by Sambrook et al. (1989). Thirty clones were recovered, and three were sequenced to check the insert identity. DNA sequencing was done by utilizing the Big Dye Terminator kit (Applied Biosystems, Foster City, CA, United States) according to the manufacturer’s recommendations and sequencing in an ABI/Prism automatic sequencer (Applied Biosystems, Foster City, CA, United States). The nucleotide sequence of *P. carvalhoi* histone H3 (GenBank access number MT508594) was 99% similar to that of *X. tropicalis* available in GenBank (DQ28350).

We used the sequence of the histone H3 clone from *P. carvalhoi* as a query in genomic searches for LocalBlast in *X. tropicalis* genome (GenBank assembly accession – GCA000004195.4). The results were manually filtered using as criteria an e-value threshold of >10–4, 80% of the identify, and alignment of at least 70% of the query.

The chromosome mapping of the H3 probe in *P. carvalhoi* karyotype was performed by FISH experiments. The plasmid mini-preparations were used as a template for probe production using PCR labeling with 11-digoxigenin-dUTP and detected with anti-DIG-Rhodamine. The hybridization experiments were conducted following the protocol of Traut et al. (2001), with minor modifications.

### Microsatellite Mapping in *P. carvalhoi* and *X. tropicalis*

The microsatellites (CA)_15_, (GA)_15_, (GATA)_8_, (CGC)_10_, (GAA)_10_, (CAG)_10_, and (GACA)_4_ were mapped to chromosome spreads of *P. carvalhoi* and *X. tropicais* using oligonucleotide probes marked directly with Cy5-fluorochrome at the 5′ end during synthesis (Sigma-Aldrich). Fluorescent *In Situ* Hybridization (FISH) assays were run following the protocol of Kubat et al. (2008) under high stringency (77%) conditions.

### Comparative Analysis of Images

The FISH experiments were run in duplicate, with the metaphases of both species being included in each run. Images of the hybridized metaphase chromosomes were captured with an Olympus BX51 Fluorescence Microscope, and the acquisitions of the images were made considering the same exposure time for the two species.

## Results

**FIGURE 1:**
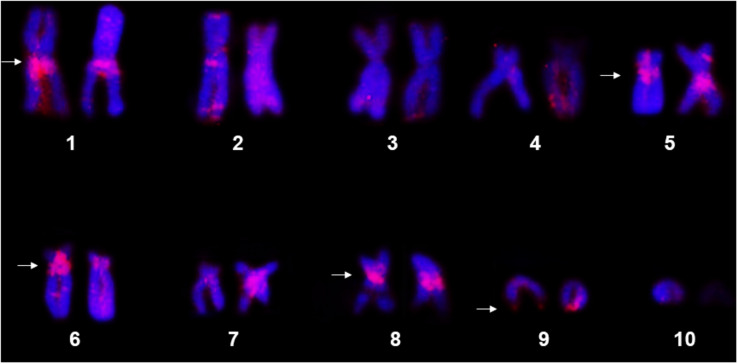
Metaphase chromosomes of *Pipa carvalhoi* submitted to fluorescent *in situ* hybridization with the histone H3 probe. The chromosome pairs with hybridization signals detected in both chromatids of each homolog are indicated by the arrowheads.

The FISH assays using the *P. carvalhoi* histone H3 probe detected hybridization signals primarily in the pericentromeric region of the short arms of pair 6 and in the long arms of the homologs of pairs 1, 5, and 8, in addition to the subterminal region of the long arms of pair 9 (Figure 1). In *X. tropicalis*, the genomic assays recovered hits with high identities (90–100%) and coverage with the histone H3 of *P. carvalhoi*, with copies being mapped in pairs 3, 6, 8, and 9 but only a few hits in pairs 2 and 5 (Supplementary Material – S1). Due to an ancient event of allotetraploidy in the origin of *X. laevis*, two subgenomes from progenitors with distinct diploid numbers evolved asymmetrically in this species, with reduced recombination, and are referred to as subgenomes L (long chromosomes) and S (short chromosome). Our genomic searches in *X. laevis* recovered only a few hits in chromosomes 5S and 5L and one hit in 6S (Supplementary Material – S1).

**FIGURE 2:**
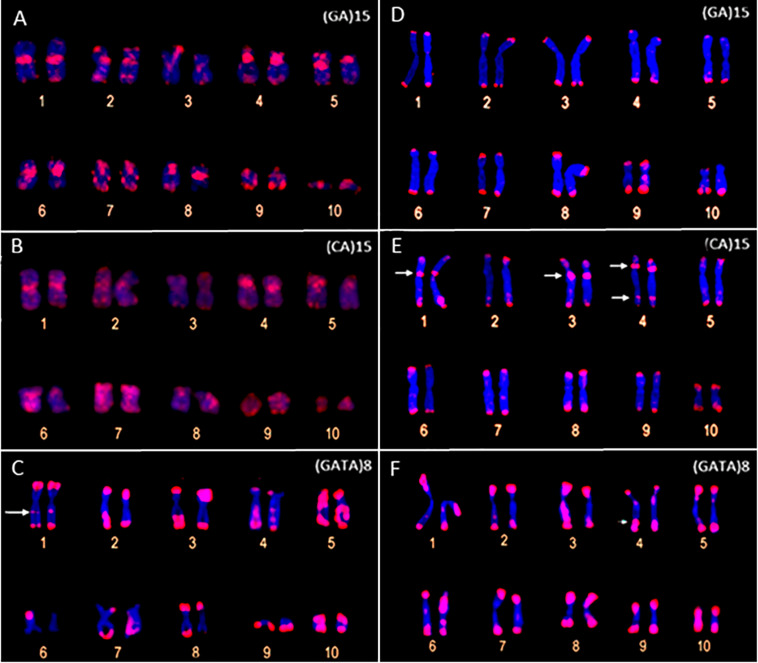
Metaphase chromosomes of *Pipa carvalhoi* **(A–C)** and *Xenopus tropicalis* **(D–F)** submitted to fluorescent *in situ* hybridization with probes for the microsatellite repeat motifs (GA)_15_ **(A,D)**, (CA)_15_ **(B,E)**, and (GATA)_8_ **(C,F)**. The arrows indicate the minor hybridization signals detected in the non-terminal regions of the chromosomes.

The FISH assays with the microsatellite probes revealed a distinct composition of the repeat motifs that compose the centromeric/pericentromeric regions of the chromosomes of *P. carvalhoi* and *X. tropicalis*. In *P. carvalhoi*, the (GA)_15_ probe detected strong hybridization signals in the centromeric region of homolog pairs 1, 2, 3, 5, 6, 7, and 8, as well as in the subterminal region of pairs 2 and 9 (Figure 2A and Table 1). In contrast with the karyotype of *X. tropicalis*, the (GA)_15_ probe detected signals in the subterminal regions in all the chromosomes of the complement (Figure 2D and Table 1). The (CA)_15_ probes detected the strongest signals in *P. carvalhoi* (Figure 2B) in the centromeric regions of chromosomes 1, 2, and 4 and the terminal regions of the telocentric chromosome 9, with weaker signals in chromosomes 5, 6, 7, and 8. In *X. tropicalis* (Figure 2E), signals of these repeats were detected in the terminal regions of most of the chromosomes of the karyotype in addition to pericentromeric signals in the chromosomes of pairs 1, 3, and 4 and some interstitial signals in the long arms of chromosome 4.

**TABLE 1:** Summary of the hybridization signal detected in *Pipa carvalhoi* and *Xenopus tropicalis* chromosomes with microsatellite probes.

	***Pipa carvalhoi***										***Xenopus tropicalis***									
**Repeat motif**	**1**	**2**	**3**	**4**	**5**	**6**	**7**	**8**	**9**	**10**	**1**	**2**	**3**	**4**	**5**	**6**	**7**	**8**	**9**	**10**
(CAG)_10_	ter	ter + Per	ter	ter	ter	ter + cen	ter	ter	ter	ter	ter	ter	ter	ter	ter	ter	ter + per	ter	ter	ter
(CGC)_10_	ter + cen	ter	ter	cen + ter	ter	ter + int	ter + int	per	ter	ter	ter	ter	ter	ter	ter	ter	ter	ter	ter	ter
(GATA)_8_	Ter + Per	ter	ter	ter + int	ter + per	ter	ter	ter	ter	ter	ter	ter	ter + int	ter + int	ter	ter + int	ter	ter	ter	ter
(GA)_15_	cen	cen + st	cen	cen + ter	cen	cen	cen + ter + int	cen	st	ter	ter	ter	ter	ter	ter	ter	ter	ter	ter	ter
(CA)_15_	cen	cen	cen + ter	cen	cen + ter	Cen	cen + ter + int	ter	ter	ter	ter + per	ter	ter + per	Per + int	ter	ter	ter	ter	ter	ter
(GAA)_10_	st	Int	ter	st	per	cen	int	int	st	ter	−	−	−	−	−	−	−	−	−	−
(GACA)_4_	−	−	−	−	−	per	−	int	−	−	−	−	−	−	−	−	−	−	−	−

*Int: interstitial; St: subterminal; Cen: centromeric; Per: pericentromeric; Ter: terminal; (−) no hybridization signal.*

**FIGURE 3:**
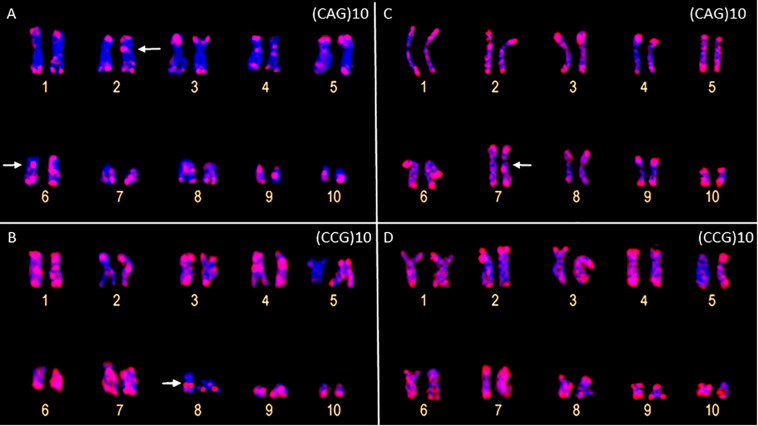
Metaphase chromosomes of *Pipa carvalhoi* **(A,B)** and *Xenopus tropicalis* **(C,D)** submitted to fluorescent *in situ* hybridization with probes for the microsatellite repeat motifs (CAG)_10_ **(A,C)** and (CCG)_10_ **(B,D)**. The arrows indicate the minor hybridization signals detected in the non-terminal regions of the chromosomes.

The FISH assays revealed a similar pattern of preferential accumulation of microsatellite motifs (CAG)_10_, (CGC)_10_, and (GATA)_8_ in the subterminal/terminal regions of all the chromosomes of *P. carvalhoi* and *X. tropicalis* (Figure 3 and Table 1). The (CAG)_10_ probe detected hybridization signals in the pericentromeric region of the long arms of pair 2 and the centromeric region of pair 6 in *P. carvalhoi* (Figure 3A), while in *X. tropicalis*, this probe detected the accumulation of signals in the pericentromeric region of the long arms of pair 7 (Figure 3D). The probe of the (CCG)_10_ motif detected a centromeric signal in the homologs of pairs 1 and 4 in *P. carvalhoi*, as well as strong signals in the pericentromeric region of pair 8 (Figure 3B), while in *X. tropicalis*, signals were detected only in the terminal regions of all the chromosomes (Figure 3E). The (GATA)_8_ probe also detected an interstitial signal in the long arms of pair 1 in *P. carvalhoi* (Figure 2C) and in the long arms of pair 4 in *X. tropicalis* (Figure 2F).

**FIGURE 4:**
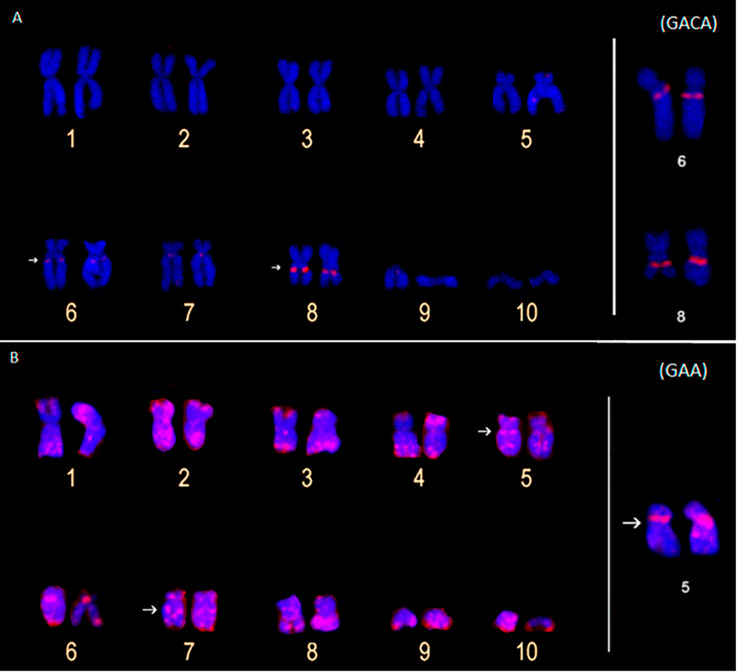
Metaphase chromosomes of *Pipa carvalhoi* submitted to fluorescent *in situ* hybridization with probes for the microsatellite repeats (GACA)4 **(A)** and (GAA)10 **(B)**. The arrows indicate the minor hybridization signals detected in the non-terminal regions of the chromosomes.

The (GACA)_4_ and (GAA)_10_ probes only detected hybridization signals in the *P. carvalhoi* chromosomes. The (GAA)_10_ probe detected signals in the subterminal regions of chromosome pairs 4 and 9, in the pericentromeric region of the homologs of pair 5, and interstitially in the long arms of pair 7 (Figure 4B). The (GACA)_4_ probe revealed an accumulation of this repeat in the pericentromeric region of the long arms of pair 6 in addition to a repetitive block in the interstitial region of the homologs of pair 8 (Figure 4A).

## Discussion

The chromosomal mapping of the repetitive DNAs in the karyotypes of *P. carvalhoi* and *X. tropicalis* expands our capacity to recognize karyological features that cannot be discerned using classical cytogenetics methods. From an evolutionary perspective, these new chromosome markers reinforce their potential for inferences on interspecific chromosomal homologies (Mezzasalma et al., 2015), and the mapping of the sequences of the histone H3 gene and the microsatellites have proven to be excellent chromosomal markers for this purpose. For example, chromosome pairs 5 and 6 bear the histone H3 cluster in *P. carvalhoi*, *X. tropicalis*, and *X. laevis*, indicating a long history of conservation in the chromosome-bearing histone locus, given that *P. carvalhoi* and *Xenopus* are estimated to have diverged at least 136 million years ago (Cannatella, 2015). A similar scenario was also observed in chromosome pair 5 of the three species, even though fewer sequences were found in the *X. tropicalis* chromosomes.

Another interesting finding of the present study was the presence of histone H3 copies in the homologs of pairs 8 and 9 in the *P. carvalhoi* and *X. tropicalis* chromosomes, whereas this signal was absent in these chromosomes in *X. laevis*. While *P. carvalhoi* and *X. tropicalis* retain 2*n* = 20 chromosomes, the putative plesiomorphic condition in the Pipidae (Mezzasalma et al., 2015), *X. laevis* has a diploid number of 2*n* = 36, which originated from a process of allopolyploidy, derived from the hybridization of diploid progenitors with 2*n* = 18, which are now extinct (Session et al., 2016). The complex evolutionary history of *X. laevis* involved the subsequent doubling of the genome to restore meiotic pairing (Session et al., 2016). The 2*n* = 18 karyotype would have originated by 9 + 10 *in tandem* fusion in the ancestral lineages of *X. laevis* (see Mezzasalma et al., 2015). Thus, chromosomes bearing the histone H3 copies that are shared between *X. tropicalis* and *P. carvalhoi* corroborate the hypothesis that 2*n* = 20 is the primitive diploid number of the Pipidae and that these two species retain more than simple numerical and morphology similarities (Mezzasalma et al., 2015) but may also share the gene content of their chromosomes. *X. laevis* may have lost these markers (or they have degenerated) during its genomic reorganization and the allopolyploidy process, which resulted in the conservation only of the copies in chromosome pairs 5 (in both subgenomes – 5S and 5L) and 6S.

The conservation of chromosomes that bear histone clusters has already been reported in other groups (Cabrero et al., 2009; Cabral-De-Mello et al., 2011; Mandrioli and Manicardi, 2013; Traldi et al., 2019) and reflects the strong selective pressures acting on these markers. As these markers are also highly conserved in *X. tropicalis* and *P. carvalhoi*, the physical mapping of this sequence in other pipid species should provide more evidence to support this evolutionary hypothesis.

Our data on the chromosomal distribution of the microsatellite motifs also provide useful markers for comparative genomics at the chromosome level between *P. carvalhoi* and *X. tropicalis*. This offers new insights into the evolution of the karyotypes of these species. Our findings reveal shared patterns in the distribution and abundance of the repetitive DNA shared by these two species, such as the arrangement of the repetitive tri-[(CAG)_10_; (CGC)_10_] and tetra-nucleotide [(GATA)_8_] motifs mapped in the terminal/subterminal regions of the chromosomes, which reflects the conservation of the organization of these chromosomal regions. The enrichment of the microsatellite motifs in the terminal/subterminal region is a common phenomenon in the karyotypes of birds (Oliveira et al., 2017), fish (Cioffi et al., 2011; Xu et al., 2013; Poltronieri et al., 2014), and grasshoppers (Ruiz-Ruano et al., 2015) and may play a fundamental role in the stabilization and functioning of these chromosomal regions (Torres et al., 2011; Tashiro et al., 2017).

By contrast, the microsatellite probes detected distinct arrangements of the repetitive DNA in the chromosomes of the two species, enabling the longitudinal differentiation of pairs that are identical in their size and morphology, such as pairs 1, 2, 4, and 5. For example, the metacentric pair 1 presents pericentromeric accumulation of the (GATA)_8_ motif in the long arm of *P. carvalhoi*, which is absent in *X. tropicalis*, whereas this chromosome presents conspicuous accumulation of the (CA)_15_ motif in the pericentromeric region in the short arm in *X. tropicalis*. Chromosome pair 4 presents an exclusive accumulation signal of repeat motifs (GATA)_8_ and (GA)_15_ in the *X. tropicalis* karyotype, whereas the homologs of pair 5 exhibit exclusive hybridization signals of the (GAA)_10_ and (GATA)_8_ repeat motifs in *P. carvalhoi.* These findings highlight the unique patterns in the chromosomal organization of the repetitive DNAs in these karyotypes and their contribution to the chromosomal diversification of the two study species.

Although a few studies have evaluated the chromosomal organization of microsatellites in anuran species (Peixoto et al., 2015, 2016; Ernetti et al., 2019), we verified the clustered distribution pattern of these elements in the karyotypes of both *P. carvalhoi* and *X. tropicalis*, with clear evidence for the species-specific accumulation and distribution of some of these markers. The microsatellite repeats appear to have an intragenomic “life cycle” that includes (i) their birth in the respective genome, (ii) the subsequent increase in the number of repeats (by polymerase slippage), representing “adulthood,” and (iii) death, when the locus degrades the number of repeats by substitutions or inserts/deletions, causing the interruption of the repetitive units (Charlesworth et al., 1994; Kelkar et al., 2020). The repetitive content of the genome is thus transmitted vertically from the Most Recent Common Ancestor (MRCA) of the two species and may either remain conserved in the divergent groups or fluctuate independently in each species or lineage through the influence of stochastic mechanisms, such as polymerase slippage and recombination. These processes may also generate distinct profiles of repetitive DNA in the different lineages, as observed in the present study. Here, we evaluated only the chromosomal organization of the microsatellites by FISH assay, which limits the identification of microsatellites below the threshold of detectability by this technique (i.e., 1.5 kb).

In the present study, the clearest differences between the karyotypes were identified by the (CA)_15_ and (GA)_15_ motifs, which revealed distinct centromeric arrangements in the two study species. For example, these two repeat motifs marked all the centromeric/pericentromeric portions of the karyotype of *P. carvalhoi*, whereas in *X. tropicalis*, only pairs 1 and 3 presented clusters of (CA)_15_. The composition of the repetitive DNA that compiles the centromeric region of the chromosomes may vary considerably between closely related species (Plohl et al., 2008, 2014; Melters et al., 2013). Melters et al. (2013) analyzed 282 species of plants and animals and identified a high degree of inter- and intra-genomic variation in the composition of the repetitive centromeric DNA, which was composed primarily of long arrangements of satellite DNA and/or remnants of mobile genetic elements (Plohl et al., 2014). When present in the centromeric portion, then, the microsatellite repeats may represent a repetitive motif contained within the larger monomeric units that make up the satellite DNA of this region rather than being its principal component. Given this, our results reinforce the hypothesis of the independent evolution of the centromeric DNA content in the vertebrate karyotype that generates the species-specific profile of the repetitive DNA in this region during chromosomal evolution (Hartley and O’Neill, 2019).

We also found evidence of microsatellite enrichment in the pericentromeric region of chromosome pairs 6, 8, and 9 in the *P. carvalhoi* karyotype, which may have played a role in the rearrangements that occurred in these pairs during the diversification of the karyotype of this species. Pericentromeric inversions are the most parsimonious hypothesis to account for the chromosomal changes between the inferred primitive pipid karyotype (which is conserved in *X. tropicalis*) and *P. carvalhoi* (Mezzasalma et al., 2015). The presence of the ITSs reported by Zattera et al. (2019) and our new chromosome markers [(GA)_15_, (GACA)_4_, (CAG)_10_, and (GA)_15_ motifs] reinforce these hypotheses.

While recombination and amplification events are a major source of chromosomal variation in the density and composition of microsatellites in the genome, we cannot overlook the role of the transposable elements in the accumulation and spread of the microsatellite motifs in the genomes of these two species during the diversification of their chromosomes (Garrido-Ramos, 2017). In fact, one-third of the *X. tropicalis* genome is composed of transposable elements (Hellsten et al., 2010), and a recent draft assembly of the *P. carvalhoi* genome has also revealed numerous copies of the transposable elements in the enrichment of the microsatellite motifs (Bruschi, personal communication). Future studies that evaluate the interaction between microsatellites and transposable elements will be important for the more systematic understanding of the origins of the chromosome variation in these species.

## Conclusion

Our results have added important chromosome markers to the evolutionary comparisons of the pipid karyotypes and have corroborated inferences on the interspecific chromosomal homeologies between *P. carvalhoi* and *X. tropicalis*. This contributes to a better understanding of the chromosome changes that have occurred during the karyotypic diversification of these species. We have shown the evolutionary conservation of the chromosomes that bear the histone H3 copies and the distribution of the microsatellite motifs for at least 136 million years, the estimated time to the TMRCA of the two study species. Our data also provide clear evidence of the distinct profiles of the distribution and density of microsatellite motifs between these species, which reveals the fundamental role of these repetitive DNAs in the shaping of chromosome structure in these species. Our findings reinforce the role of the repetitive DNA in the remodeling of the karyotype architecture and indicate that the understanding of the evolutionary dynamics of these sequences in the chromosomes can increase our capacity to discriminate chromosome pairs that are identical in classical cytogenetic analyses, and consequently, our capacity to understand the mechanisms operating during chromosomal evolution of the Pipidae, in particular, *P. carvalhoi* and *X. tropicalis*.

## Data Availability Statement

All datasets presented in this study are included in the article/Supplementary Material.

## Ethics Statement

The animal study was reviewed and approved by SISBIO/Chico Mendes Institute for Biodiversity Conservation (protocol 55481-1). Written informed consent for participation was not obtained from the owners because SISBIO/Chico Mendes Institute for Biodiversity Conservation (protocol 55481-1).

## Author Contributions

MZ conducted the experiments, analyzed the data, and wrote the manuscript. CG and AS assisted in the execution and analysis of FISH experiments. TG, NP, and SR-P helped to draft the manuscript. DB designed and coordinated the study and wrote the manuscript. All authors read and approved the final version.

## Conflict of Interest

The authors declare that the research was conducted in the absence of any commercial or financial relationships that could be construed as a potential conflict of interest.
