# Supplementary material for: Evolutionary Dynamics of the Repetitive DNA in the Karyotypes of Pipa carvalhoi and Xenopus tropicalis (Anura, Pipidae)
Source: Front Genet. 2020 Jul 21;11:637. doi: 10.3389/fgene.2020.00637 (PMC7385237; doi:10.3389/fgene.2020.00637)
Supplement: Supplementary file 1 [file Table_1.DOCX]

**Table S1:** Genomic searches in *X. tropicalis* and *X. laevis* genomes using as query sequence probe of the histone H3 obtained from *P. carvalhoi*. The hits with best alignment, identity (% id) and e-value are shown.

| **Genomic position** |  | **subject** | **%id** | | ***E-*value** | |  |
| --- | --- | --- | --- | --- | --- | --- | --- |
| *Xenopus tropicalis* | | | | | | | |
| **Chromosome 2** | NC_030678.1 | | | 92.33 | | 5,00E-125 | |
|  | NC_030678.1 | | | 92.33 | | 5,00E-125 | |
|  | NC_030678.1 | | | 92.33 | | 5,00E-125 | |
| **Chromosome 3** | gi\|1049006347\|ref\|NW_016683361.1\| | | | 90.71 | | 2,00E-111 | |
|  | gi\|1049006347\|ref\|NW_016683361.1\| | | | 90.40 | | 5,00E-109 | |
|  | gi\|1049006347\|ref\|NW_016683361.1\| | | | 90.40 | | 5,00E-109 | |
|  | gi\|1049006347\|ref\|NW_016683361.1\| | | | 90.40 | | 5,00E-109 | |
|  | gi\|1049006347\|ref\|NW_016683361.1\| | | | 90.40 | | 5,00E-109 | |
|  | gi\|1049006347\|ref\|NW_016683361.1\| | | | 90.40 | | 5,00E-109 | |
|  | gi\|1049006347\|ref\|NW_016683361.1\| | | | 90.40 | | 5,00E-109 | |
|  | gi\|1049006347\|ref\|NW_016683361.1\| | | | 90.40 | | 5,00E-109 | |
|  | gi\|1049006347\|ref\|NW_016683361.1\| | | | 90.40 | | 5,00E-109 | |
|  | gi\|1049006347\|ref\|NW_016683361.1\| | | | 90.40 | | 5,00E-109 | |
| **Chromosome 5** | gi\|1049006345\|ref\|NW_016683363.1\| | | | 81.75 | | 2,00E-10 | |
| **Chromosome 6** | gi\|1049006344\|ref\|NW_016683364.1\| | | | 93.88 | | 1,00E-137 | |
|  | gi\|1049006344\|ref\|NW_016683364.1\| | | | 93.88 | | 1,00E-137 | |
|  | gi\|1049006344\|ref\|NW_016683364.1\| | | | 93.88 | | 1,00E-137 | |
|  | gi\|1049006344\|ref\|NW_016683364.1\| | | | 93.58 | | 3,00E-135 | |
| **Chromosome 8** | gi\|1049006342\|ref\|NW_016683366.1\| | | | 93.75 | | 1,00E-04 | |
|  | gi\|1049006342\|ref\|NW_016683366.1\| | | | 100.00 | | 0.12 | |
|  | gi\|1049006342\|ref\|NW_016683366.1\| | | | 100.00 | | 0.12 | |
|  | gi\|1049006342\|ref\|NW_016683366.1\| | | | 100.00 | | 1.9 | |
|  | gi\|1049006342\|ref\|NW_016683366.1\| | | | 100.00 | | 1.9 | |
|  | gi\|1049006342\|ref\|NW_016683366.1\| | | | 100.00 | | 7.3 | |
|  | gi\|1049006342\|ref\|NW_016683366.1\| | | | 100.00 | | 7.3 | |
|  | gi\|1049006342\|ref\|NW_016683366.1\| | | | 100.00 | | 7.3 | |
|  | gi\|1049006342\|ref\|NW_016683366.1\| | | | 100.00 | | 7.3 | |
|  | gi\|1049006342\|ref\|NW_016683366.1\| | | | 100.00 | | 7.3 | |
|  | gi\|1049006342\|ref\|NW_016683366.1\| | | | 100.00 | | 7.3 | |
|  | gi\|1049006342\|ref\|NW_016683366.1\| | | | 100.00 | |  | |
| **Chromosome 9** | gi\|1049006341\|ref\|NW_016683367.1\| | | | 93.27 | | 4,00E-133 | |
|  | gi\|1049006341\|ref\|NW_016683367.1\| | | | 93.27 | | 4,00E-133 | |
|  | gi\|1049006341\|ref\|NW_016683367.1\| | | | 92.97 | | 1,00E-130 | |
|  | gi\|1049006341\|ref\|NW_016683367.1\| | | | 92.97 | | 1,00E-130 | |
|  | gi\|1049006341\|ref\|NW_016683367.1\| | | | 92.66 | | 3,00E-128 | |
|  | gi\|1049006341\|ref\|NW_016683367.1\| | | | 92.64 | | 1,00E-127 | |
|  | gi\|1049006341\|ref\|NW_016683367.1\| | | | 92.33 | | 2,00E-125 | |
|  | gi\|1049006341\|ref\|NW_016683367.1\| | | | 92.33 | | 2,00E-125 | |
|  | gi\|1049006341\|ref\|NW_016683367.1\| | | | 92.33 | | 2,00E-125 | |
|  | gi\|1049006341\|ref\|NW_016683367.1\| | | | 92.33 | | 2,00E-125 | |
|  | gi\|1049006341\|ref\|NW_016683367.1\| | | | 92.33 | | 2,00E-125 | |
|  | gi\|1049006341\|ref\|NW_016683367.1\| | | | 92.33 | | 2,00E-125 | |
|  | gi\|1049006341\|ref\|NW_016683367.1\| | | | 92.33 | | 2,00E-125 | |
|  | gi\|1049006341\|ref\|NW_016683367.1\| | | | 92.33 | | 2,00E-125 | |
|  | gi\|1049006341\|ref\|NW_016683367.1\| | | | 92.33 | | 2,00E-125 | |
|  | gi\|1049006341\|ref\|NW_016683367.1\| | | | 92.33 | | 2,00E-125 | |
|  | gi\|1049006341\|ref\|NW_016683367.1\| | | | 92.33 | | 2,00E-125 | |
|  | gi\|1049006341\|ref\|NW_016683367.1\| | | | 92.33 | | 2,00E-125 | |
|  | gi\|1049006341\|ref\|NW_016683367.1\| | | | 91.44 | | 9,00E-119 | |
|  | gi\|1049006341\|ref\|NW_016683367.1\| | | | 95.97 | | 5,00E-65 | |
|  | gi\|1049006341\|ref\|NW_016683367.1\| | | | 89.90 | | 2,00E-58 | |
| *Xenopus laevis* | | | | | | | |
| **Chromosome 5L** | CM004474.1  CM004474.1  CM004474.1  CM004474.1  CM004474.1 | | | 88.07  88.07  88.07  94.34  81.73 | | 3,00E-92  3,00E-92  3,00E-92  1,00E14  7,00E-07 | |
| **Chromosome 5S** | CM004475.1  CM004475.1  CM004475.1  CM004475.1 | | | 88.07  88.07  87.77  83.33 | | 3,00E-92  3,00E-92  7,00E-90  3,00E-15  3,00E-15 | |
| **Chromosome 6S** | CM004477.1 | | | 91.74 | | 6,00E-121 | |
